# Effect of Video-Assisted Reflective Practice on Infection Control Performance During Oral Hygiene Procedures Performed by Dental Hygienists

**DOI:** 10.3290/j.ohpd.c_2491

**Published:** 2026-03-17

**Authors:** Myoung-Hee Kim, Young Sun Hwang

**Affiliations:** a Myoung-Hee Kim Professor, Department of Dental Hygiene, College of Health Science, Eulji University, Sanseong-Daero, Sujeong-Gu, Seongnam, Korea. Performed the experiments, contributed to the statistical analysis, discussed the results, proofread the manuscript, read and approved the final manuscript.; b Young Sun Hwang Professor, Department of Dental Hygiene, College of Health Science, Eulji University, Sanseong-Daero, Sujeong-Gu, Seongnam, Korea. Experimental design, performed the experiments, wrote the original draft, contributed to the statistical analysis, discussed the results, proofread the manuscript, read and approved the final manuscript.

**Keywords:** cross-infection, verbal instruction, video-assisted reflective practice.

## Abstract

**Purpose:**

The dental clinic environment is highly vulnerable to cross-infection from patients’ blood and oral fluids. Regular training is vital to prevent cross-contamination between healthcare providers and patients. This study evaluated the effectiveness of video-assisted reflective practice compared to traditional verbal Instruction for infection-control education.

**Materials and Methods:**

Dental hygienists participated in the study, during which their dental calculus removal procedures were video-recorded. Participants were randomly assigned to one of two groups: the verbal instruction group received conventional training on infection control, while the video-assisted reflective practice group reviewed video recordings of their own clinical performance with guidance from a researcher. Following the educational intervention, all participants repeated the calculus removal procedure, and this session was also recorded. The effectiveness of each instructional method was assessed by analyzing the number of non-clinical surface contacts during dental hygiene procedures in the video recordings, both before and after the intervention.

**Results:**

Video analysis revealed that both verbal instruction and video-assisted reflective practice effectively reduced non-clinical surface contact during dental hygiene procedures. However, the reduction was statistically significantly greater in the video-assisted reflective practice group. Notably, the dental unit chair and the clinician’s knee, identified as the most frequent contact sites, showed statistically significantly greater reductions in contact frequency in this group compared to the verbal instruction group.

**Conclusion:**

This study demonstrates that video-assisted reflective practice, which allows clinicians to reflect on their own behavior, is effective for infection control training. When combined with verbal instruction, it may further enhance dental hygienists’ self-directed competence.

Cross-infection refers to the transmission of pathogens from one individual to another, either directly or indirectly. The dental care environment poses a particularly high risk for cross-infection due to frequent exposure to biological media such as blood, saliva, mucous membranes, and aerosols.^15^ Transmission may occur through direct contact with blood, saliva, open wounds, or mucosa, or through indirect contact via contaminated instruments, gloves, dental unit chairs, and handpieces. Pathogens can also spread through the air via droplets or aerosols generated by speaking, coughing, or operating equipment such as ultrasonic scalers and high-speed handpieces. These infectious particles can enter the body through mucous membranes or the respiratory tract, contributing to the airborne transmission of respiratory infections, including tuberculosis and COVID-19. Self-inoculation may also occur when individuals touch their eyes, nose, or mouth with contaminated hands. Self-inoculation may also occur when individuals touch their eyes, nose, or mouth with contaminated hands. Furthermore, dental professionals who care for multiple patients may become vectors of cross-infection if hygiene practices—such as hand hygiene and proper replacement of personal protective equipment—are not adequately followed. Infected individuals, including both patients and staff, may also contribute to the spread of pathogens within the clinic or even into the surrounding community.

A notable case was reported in a dental clinic in Oklahoma, USA, where multiple patients contracted hepatitis B, hepatitis C, and HIV due to the reuse of syringes and needles and improper instrument sterilization (ABC News).^1^ In response to heightened infection control concerns following the COVID-19 pandemic, the Korean Dental Association and the Korea Disease Control and Prevention Agency established standardized guidelines to strengthen infection prevention in dental settings.^10^ These guidelines emphasize enhanced infection control measures, including the use of rubber-dams, high-volume suction devices, N95 respirators, sterilization protocols, hand hygiene, and comprehensive personal protective equipment (PPE).

Periodic infection control training is a critical strategy for enhancing the safety and reliability of dental healthcare institutions. Educational methods for infection control commonly include verbal instruction (e.g., lecture-based formats) and video-based training, which utilizes video demonstrations of clinical procedures and infection control practices.^16^ Verbal instruction typically involves question-and-answer sessions focused on types of infectious diseases, pathways of cross-infection, and standard precautions. Video-based training offers visual representations of actual clinical procedures, including sterilization techniques, proper glove application, and instrument processing protocols. It supports repeated learning and is easily accessible via mobile devices such as smartphones and tablets, without limitations of time or location. Hands-on training including clinical simulations using manikins (head and torso), personal protective equipment (PPE) application exercises, operation of sterilization equipment, and hygiene management training for dental unit chairs, can further maximize the effectiveness of both didactic and video-based education. Microbial contamination on the surfaces of dental equipment exposed to patients’ blood or saliva can serve as a reservoir for pathogenic bacteria, contributing to cross-contamination. Moreover, because aerosols containing oral microorganisms are inevitably generated during dental procedures, the level of surface bacterial contamination in dental clinics is generally high. This underscores the necessity of regular and effective infection control education to raise awareness and promote safe practices among dental professionals.^2,17^

In this study, we evaluated the effectiveness of video-assisted reflective practice, an educational approach in which dental hygienists recorded their oral hygiene procedures and reviewed their own performance to identify and reflect on infection control issues. This method allows learners to visually assess their clinical behavior and recognize areas requiring improvement. The aim of the study was to assess the utility of self-observation-based video training as a method for infection control education.

## MATERIAL AND METHODS

### Ethical Considerations

This study was reviewed and approved by the Institutional Review Board of Eulji University (approval No. EU25-002). All procedures were conducted in accordance with the ethical standards outlined in the Declaration of Helsinki. All participants were informed of the study procedures and provided written consent prior to participation.

### Participant Recruitment

Dental hygienists capable of performing dental calculus removal using scaling instruments were recruited as participants. The minimum sample size was calculated using G*Power based on an F distribution. A minimum of 15 participants per group was required, based on the following parameters: effect size (Cohen’s f) = 0.5 (medium), significance level (α) = 0.05, and statistical power (1−β) = 0.8 (80%). Taking into account potential dropouts during the experiment, 20 participants were assigned to each group.

### Manual Scaling of Dental Calculus

One day prior to the experiment, 1 g of artificial dental calculus was applied to the entire arch of a dentiform using a dental calculus simulation set (Nissin Dental Products; Tokyo, Japan). Participants mounted the dentiform onto a dental manikin and performed detection and removal of the artificial calculus using scaling instruments over a standardized duration of 20 min. The entire procedure was video-recorded for each participant.

### Verbal Instruction and Video-Assisted Reflective Practice

**Fig 1:**
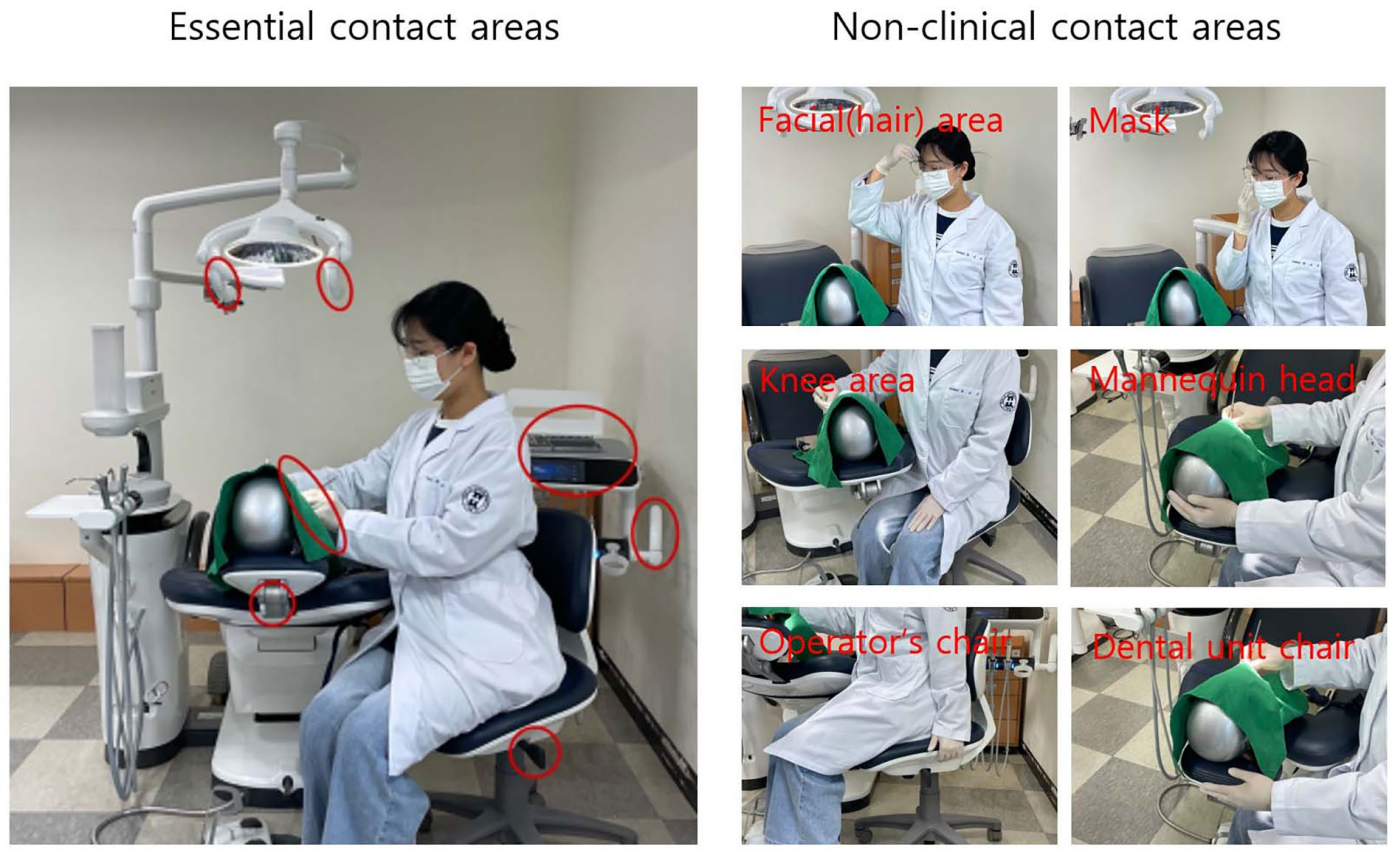
Classification of contact areas during oral hygiene procedures based on necessity of contact by the operator. Essential contact areas include the bracket table, bracket table handles, light handle, headrest adjustment handle, operator chair height adjustment lever, and dental drape. Non-clinical surface contact areas include the operator’s face, dental mask, and knee, the surface of the manikin head excluding the dental drape, the operator’s chair, and the dental unit chair. All identified areas are indicated by red circles in the image.

In this study, contact areas during dental hygiene procedures were classified as either essential or non-clinical. Essential contact areas included the bracket table, bracket table handles, light handle, headrest adjustment handle, operator chair height adjustment lever, and the dental drape. Non-clinical contact areas included the operator’s face, dental mask, and knee; the surface of the manikin head excluding the dental drape; the operator’s chair; and the dental unit chair (Fig 1). After placing the manikin with assembled dentiforms containing artificial calculus on the dental unit chair, all participants used hand instruments for 20 min to detect and remove the artificial calculus attached to the full dentition of the dentiform. Once the removal of artificial calculus was completed, the participants were conveniently divided into two groups: verbal instruction and video-assisted self-reflection. In the verbal instruction group, the researcher reviewed each participant’s recorded procedure and provided verbal explanations regarding non-clinical contact areas and the associated risks of cross-infection. In the video-assisted reflective practice group, participants reviewed their own recorded scaling procedures with the researcher, identified instances of non-clinical contact, and received similar verbal instruction on potential cross-infection risks. After the intervention, both groups repeated the calculus detection and removal procedure on the dentiform using scaling instruments for an additional 20 min. This second procedure was also video-recorded. The effectiveness of each educational method was assessed by comparing the number of contacts with non-clinical surfaces before and after the intervention, based on analysis of the video recordings.

### Statistical Analysis

Statistical analysis was conducted using SPSS version 28.0. The number of contacts with non-essential surfaces during dental hygiene procedures was presented as mean ± standard deviation (SD). The Shapiro–Wilk test indicated that the assumption of normality was not satisfied. Therefore, within-group differences between pre- and post-education were analyzed using the non-parametric Wilcoxon signed-rank test, and differences in changes between the two independent groups according to the educational method were analyzed using the non-parametric Mann-Whitney U-test. A p-value ≤ 0.05 was considered statistically significant.

## RESULTS

**Fig 2:**
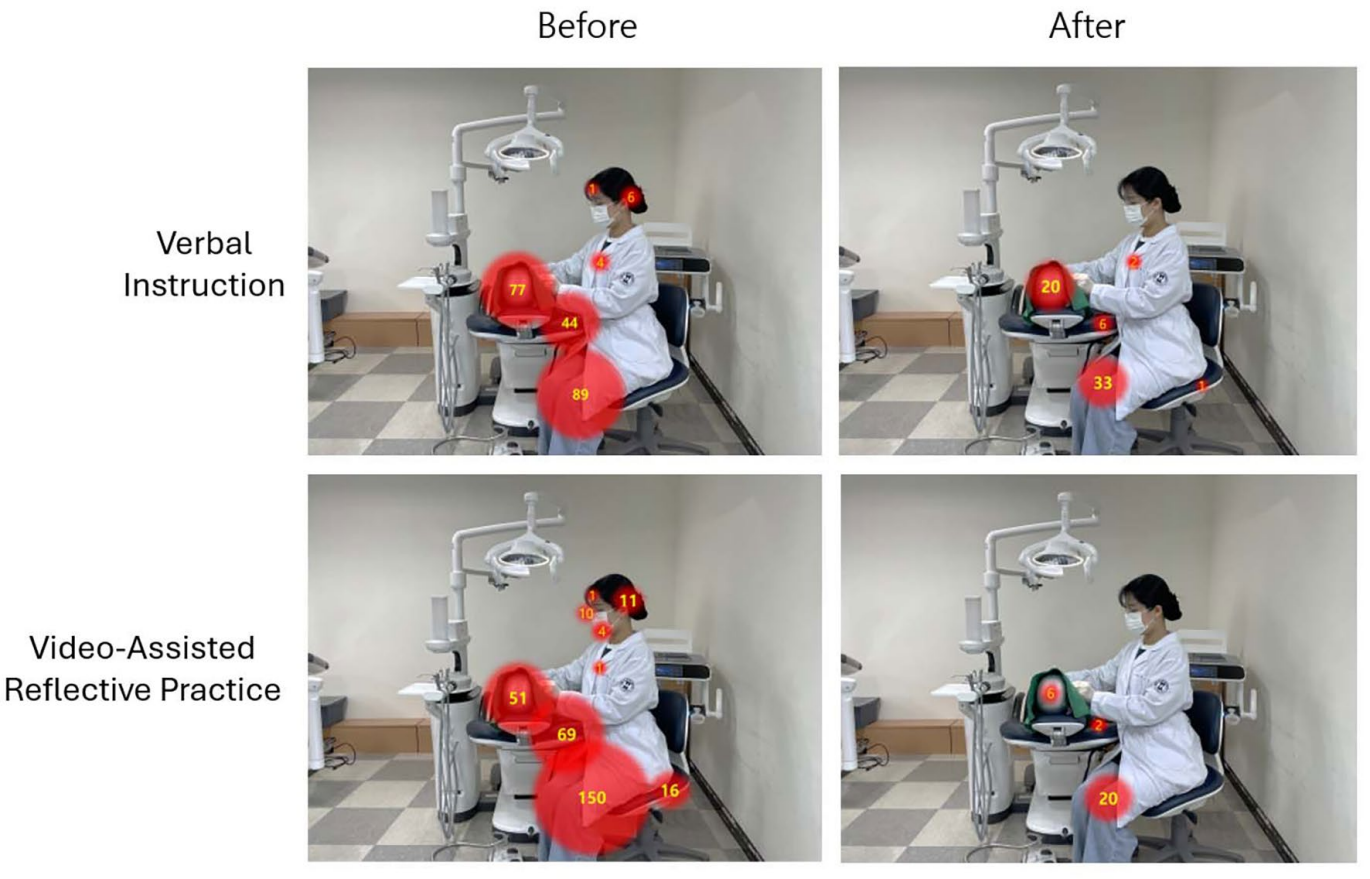

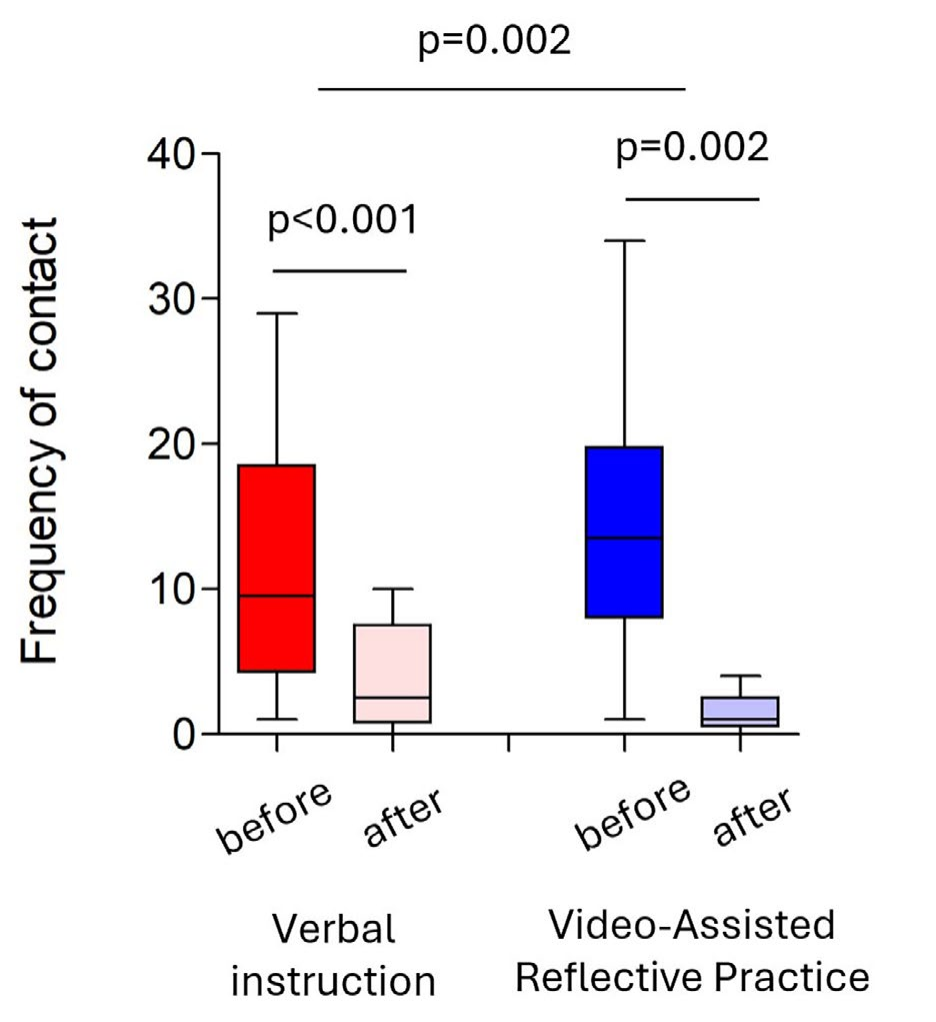
Comparison of non-clinical surface contact frequencies before and after verbal instruction video-assisted reflective practice. (a) Contact frequencies with non-clinical surfaces were identified from pre- and post-education video recordings of oral hygiene procedures in each group. Representative contact patterns for a single participant are illustrated. (b) Group-level differences in non-clinical surface contact frequencies before and after the educational interventions are presented graphically (n=20/group). Statistical significance within each group (pre- vs post-intervention) was assessed using the Wilcoxon signed-rank test. Between-group differences in pre-post change were evaluated using the Mann-Whitney U-test. p ≤ 0.05 was considered statistically significant.

To evaluate effective methods for infection control education in dental hygiene, this study compared the outcomes of verbal instruction and video-assisted reflective practice. Participants manually detected and removed artificial calculus applied to all teeth of a dentiform using scaling instruments, and the entire procedure was video-recorded. They were then randomly assigned to either the verbal instruction group or the video-assisted reflective practice group. In the verbal instruction group, the researcher reviewed each participant’s recorded procedure and provided verbal instruction, identifying non-clinical contact areas and explaining the associated risks of cross-infection. In the video-assisted reflective practice group, the participant reviewed their recorded calculus removal procedure together with the researcher, identified non-clinical contact areas, and received verbal instruction on potential cross-infection risks. After the educational interventions were completed, both groups repeated the calculus detection and removal procedures on the dentiform under identical conditions, and these sessions were also recorded. The effectiveness of each educational method was assessed by counting the number of contacts with predefined non-clinical surfaces including the operator’s face, dental mask, and knee; the surface of the manikin head (excluding the dental drape); the operator’s chair; and the dental unit chair, before and after the intervention (Fig 2a). The analysis revealed that both verbal instruction (p < 0.001) and video-assisted reflective practice (p = 0.002) statistically significantly reduced the number of contacts with non-clinical surfaces (Fig 2b). These results indicate that both educational approaches effectively improved infection control performance during dental hygiene procedures. However, comparison of the magnitude of change between groups showed that video-assisted reflective practice resulted in a statistically significantly greater reduction in non-clinical surface contact than verbal instruction (p = 0.002).

The effectiveness of each educational method was further evaluated by focusing on the three areas with the highest frequency of non-clinical contact: the manikin head, the dental unit chair, and the operator’s knee (Table 1). For the manikin head, the frequency of contact statistically significantly decreased following verbal instruction, from 3.9 ± 3.5 to 1.0 ± 1.9 (p < 0.001). A similar statistically significant reduction was observed in the video-assisted reflective practice group, from 2.7 ± 3.2 to 0.3 ± 0.7 (p < 0.001). For the dental unit chair, the verbal instruction group showed a reduction in contact frequency from 2.2 ± 3.9 to 0.3 ± 1.7; however, this change was not statistically significant (p = 0.057). In contrast, the video-assisted reflective practice group demonstrated a statistically significant decrease from 4.7 ± 6.5 to 0.1 ± 0.5 (p < 0.001). For the operator’s knee, contact frequency in the verbal instruction group declined from 4.5 ± 7.4 to 1.7 ± 2.6, but this reduction was not statistically significant (p = 0.116). The video-assisted reflective practice group, however, showed a statistically significant decrease from 7.5 ± 5.8 to 1.0 ± 3.4 (p < 0.001). Among the three areas, only the operator’s knee exhibited a statistically significant difference between the two educational methods in terms of change in contact frequency before and after the intervention (p = 0.043).

**Table 1:** Comparison of contact frequencies according to educational method (n=20/group)

Contact sites	Educational method	Contact frequency (mean±SD)		p-value	
Mannequin head	Verbal instruction	before	3.9±3.5	<0.001	0.529
		after	1.0±1.9		
	Video-assisted reflective practice	before	2.7±3.2	<0.001	
		after	0.3±0.7		
Dental unit chair	Verbal instruction	before	2.2±3.9	0.057	0.068
		after	0.3±1.7		
	Video-assisted reflective practice	before	4.7±6.5	0.001	
		after	0.1±0.5		
Knee area	Verbal instruction	before	4.5±7.4	0.116	0.043
		after	1.7±2.6		
	Video-assisted reflective practice	before	7.5±5.8	<0.001	
		after	1.0±3.4		
Wilcoxon’s signed-rank test; difference in frequency of contact before and after, Mann-Whitney U-test; difference between verbal instruction and video-assisted reflective practice.					

## DISCUSSION

Microbial contamination of dental equipment surfaces exposed to patients’ blood and saliva can serve as a reservoir for bacteria and may contribute to cross-contamination.^15^ The detection of substantial levels of oral streptococci, Pseudomonas spp., and Staphylococcus aureus from the internal components of dental handpieces that had not been sterilized underscores the critical role of dental equipment as a potential reservoir of pathogens.^12^ The presence of oral microorganisms in dental clinics is a major cause of contamination and infection in dental settings. In a study evaluating bacterial contamination across five departments in a dental hospital (pediatric dentistry, implantology, prosthodontics, oral medicine, and restorative dentistry) various surfaces were found to harbor bacterial species. These included dental chair armrests, sinks and faucets, floors beneath dental chairs, towel dispensers, handles connected to lights and instrument trays, dental records, x-ray viewers, benches, and headrests. The identified bacterial species included Staphylococcus, Streptococcus, Pseudomonas, Bacillus, and Micrococcus.^2,7^ The bacterial load on surfaces in dental operatories was reported as follows: 44.82 × 10^3^ CFU/ml on cuspidors, 5.47 × 10^3^ CFU/ml on light handles, and 16.28 × 10^3^ CFU/ml on dental unit chairs, with cuspidors showing the highest levels of contamination.^17^ Contamination levels were higher in larger institutions (i.e., hospital-level clinics) and increased proportionally with the number of patients treated.^4,17^ Regarding microbial identification, Gram-positive bacteria accounted for 47.3% and Gram-negative bacteria for 52.7% of all isolates. Among the Gram-positive species, Micrococcus luteus (10.9%), Bacillus pumilus, and Staphylococcus aureus were detected. Among the Gram-negative isolates, species such as Acinetobacter ursingii, Brevundimonas diminuta, Chryseobacterium (Flavobacterium) indologenes (CDC IIb), and Methylobacterium spp. were identified.^4,11^ This finding demonstrates the predominance of Gram-negative bacteria in the dental environment and suggests that the clinical setting may serve as a potential reservoir of infection. It underscores the necessity of comprehensive infection control measures throughout the dental clinic. Nevertheless, recent evaluations have reported that various clinical surfaces in dental school settings remain heavily contaminated with microorganisms, including Staphylococcus aureus.^11^ Furthermore, it has been reported that dental hygienists’ uniforms are typically laundered only two to three times per week, or in some cases less than once per week.^13^ Even among dental students who are expected to receive comprehensive infection control training, high awareness of microbial contamination of clinical gowns has been reported, yet concern regarding the potential for cross-contamination via gowns remains relatively low.^5^

This study aimed to identify an effective method of infection control education for dental hygienists, with the goals of enhancing learner engagement, improving educational outcomes, strengthening infection control performance, and ultimately reducing the risk of cross-infection. Specifically, we sought to evaluate which of two commonly used methods, verbal instruction or video-based education, would be more effective for infection control training. Findings from systematic reviews and meta-analyses have demonstrated that video-based learning provides opportunities for repeated practice and greater clarity compared to live demonstrations, resulting in statistically significantly improved educational outcomes in both skill acquisition and knowledge gain.^3^ Based on this evidence, we sought to explore the effectiveness of video-assisted self-reflection as a method of dental infection control education and compared it with conventional verbal instruction. Importantly, this study focused on video-based education not as a conventional one-way instructional tool, but as a self-observation-centered method, comparing its effectiveness with that of traditional verbal instruction. In the self-observation-based video education group, dental hygienists reviewed video recordings of their own oral hygiene procedures to voluntarily recognize deficiencies in their infection control practices. By visually identifying unconscious behavioral errors, participants were encouraged to make self-directed improvements. Although both conventional video-based instruction and self-observation-based video training utilize audiovisual materials, they differ substantially in educational purpose and learning impact. Traditional video instruction is primarily designed for knowledge transfer, where learners passively observe expert demonstrations and replicate the modeled behaviors. While effective for introducing new tasks, this method often promotes passive learning. In contrast, video self-reflection or video feedback training involves learners observing recordings of their own clinical performance, which fosters behavior modification, practical skill development, and critical thinking. This approach encourages self-awareness, reflective learning, and active engagement.^8,9^ In this study, we examined the impact of video self-observation training on the infection control performance of dental hygienists, a group for whom heightened awareness of cross-contamination risk is essential. Our findings confirmed that verbal instruction was effective in reducing non-clinical surface contact. However, self-observation-based video training was also effective, and notably more effective than verbal instruction in reducing contact frequency with the operator’s knee, one of the most frequently contacted non-clinical surfaces during dental hygiene procedures.

Dental healthcare professionals routinely receive infection control training and are generally aware of the vulnerability of clinical dental environments to infection. To prevent cross-contamination during oral treatment procedures, surfaces that require contact are typically covered with protective materials such as synthetic resin barriers, aluminum foil, or disposable non-woven fabrics.^6^ In addition, clinicians wear personal protective equipment (PPE), including gloves, gowns, and masks, and surrounding surfaces are disinfected to minimize infection risk.^6,14^ Despite these proactive measures, the emergence of infectious diseases, particularly respiratory pandemics such as COVID-19, has underscored the need for more effective infection control education. In this study, we found that behavior-observation-based video training was more effective than traditional verbal instruction, which primarily relies on one-way information delivery. Specifically, this approach statistically significantly reduced contact frequency with three surfaces most vulnerable to infection control lapses during dental hygiene procedures: the manikin head, the dental unit chair, and the operator’s knee. This training method appears well-suited not only for educating new personnel entering clinical practice due to its clarity and use of visual feedback but also for continuing professional education. By promoting self-reflection through direct observation and analysis of one’s own clinical behavior, behavior-observation-based video training enables individuals to identify and improve weaknesses in their infection control practices.

Using a sample size calculation program, we compared the two educational methods based on the analysis of 20 participants per group. For this purpose, artificial plaque attached to dentiforms assembled on a manikin head was used, and non-clinical surface contact during dental hygiene procedures was observed. However, in actual patient care settings, the frequency and complexity of such contacts are likely to be considerably higher. Unlike manikin heads, which remain fixed in position and angle, human oral anatomy varies statistically significantly in dental arch morphology, buccolingual space, and soft tissue characteristics (e.g., tongue, cheeks, palate). Additionally, dynamic factors such as jaw repositioning and soft-tissue displacement frequently occur during treatment, resulting in more complex contact interactions than those observed in simulation-based environments. Further complicating real-world procedures are variables such as patient cooperation, movement, salivary flow, and involuntary soft tissue responses, all of which often require the use of adjunctive tools such as oral irrigators, suction devices, cheek retractors, and saliva ejectors — factors not present in the simulated environment. As such, the contact frequency measured using manikins may underestimate the actual frequency of contact in clinical practice. Another limitation of this study is the exclusive use of scaling instruments to remove artificial calculus from dentiforms. In clinical practice, ultrasonic scalers are more commonly employed due to their efficiency and patient comfort. We confirmed that the educational effect of video-assisted reflective practice was statistically significant in specific aspects. However, further studies with larger sample sizes, conducted in real clinical settings with ultrasonic scalers, are needed to evaluate the effectiveness of self-reflection–based video education in infection control practices.

## CONCLUSION

The dental clinic is a high-risk environment with substantial potential for cross-infection, emphasizing the need for rigorous infection control measures. Video-assisted reflective practice provides practitioners with an effective tool to objectively evaluate their infection control performance and to identify and improve vulnerable areas. Thus, video-assisted reflective practice education not only reinforces the self-directed infection control competence of dental hygienists but also highlights its considerable value as an educational approach for infection control in dentistry.

## ACKNOWLEDGEMENTS

We would like to thank the “Pine Tree Club” of the Department of Dental Hygiene at Eulji University for their valuable support in this research. This research was supported by Basic Science Research Program through the National Research Foundation of Korea (NRF) funded by the Ministry of Education, Science and Technology (2022R1F1A1063204).

## REFERENCES
